# Towards the description of livestock mobility in Sahelian Africa: Some results from a survey in Mauritania

**DOI:** 10.1371/journal.pone.0191565

**Published:** 2018-01-24

**Authors:** Andrea Apolloni, Gaëlle Nicolas, Caroline Coste, Ahmed Bezeid EL Mamy, Barry Yahya, Ahmed Salem EL Arbi, Mohamed Baba Gueya, Doumbia Baba, Marius Gilbert, Renaud Lancelot

**Affiliations:** 1 French Agricultural Research and International Cooperation Organization for Development (Cirad), Department of Biological Systems (Bios), UMR Animals, Health, Territories, Risks, and Ecosystems (Astre), Campus International de Baillarguet, 34398 Montpellier, France; 2 French National Agricultural Research Center for International Development, Animal Health Department, UMR Astre, Campus International de Baillarguet, 34398 Montpellier, France; 3 Institut Sénégalais de Recherches Agricoles, Laboratoire National d’Elevage et de Recherches Vétérinaires (LNERV), route du Front de Terre, BP 2057 Dakar-Hann, Sénégal; 4 Université Libre de Bruxelles, Spatial epidemiology Lab., 1050 Brussels, Belgium; 5 Office National de Recherches et de Développement de l’Elevage (ONARDEL), BP 167 Nouakchott, Mauritania; 6 Ministere de l’Elevage, BP 167 Nouakchott, Mauritania; 7 Fonds National de la Recherche Scientifique, 1000 Brussels, Belgium; Universidad Rey Juan Carlos, SPAIN

## Abstract

Understanding spatio-temporal patterns of host mobility is a key factor to prevent and control animal and human diseases. This is utterly important in low-income countries, where animal disease epidemics have strong socio-economic impacts. In this article we analyzed a livestock mobility database, whose data have been collected by the Centre National d’Elevage et de Recherches Vétérinaires (CNERV) Mauritania, to describe its patterns and temporal evolution. Data were collected through phone and face-to-face interviews in almost all the regions in Mauritania over a period of roughly two weeks during June 2015. The analysis has shown the existence of two mobility patterns throughout the year: the first related to routine movements from January to August; the second strictly connected to the religious festivity of Tabaski that in 2014 occurred at the beginning of October. These mobility patterns are different in terms of animals involved (fewer cattle and dromedaries are traded around Tabaski), the means of transportation (the volume of animals moved by truck raises around Tabaski) and destinations (most of the animals are traded nationally around Tabaski). Due to the differences between these two periods, public health officers, researchers and other stakeholders should take account of the time of the year when implementing vaccination campaigns or creating surveillance networks.

## Introduction

Livestock trade is one of the major factors contributing to the spread of animal and zoonotic diseases. Consequently, understanding livestock mobility patterns constitutes a fundamental step to prevent and control epidemics. In Europe and the U.S.A., livestock trade databases, created by national or regional agencies, can be used by researchers to describe mobility patterns, optimize disease surveillance and control [1], and predict possible epidemic scenarios [2–8]. Other countries are trying to improve the identification and traceability of animal movements as suggested by the World Animal Health Organization (OIE)’s International Animal Health Code [9].

Sahelian Africa encompasses countries located between the Atlantic Ocean (west and south), the Sahara Desert (north), and sub-humid / humid Africa (south). In this region, most of the livestock trade is made of live animals (as opposed to simply their derived meat), and transhumance/nomadism is widespread. Indeed, local livestock production systems are based on animal mobility to optimize the access to water and forage resources [10, 11]. Animals are herded towards best quality grazes [12] either throughout the year (nomadism) or during specific periods (transhumance) [13, 14]. This search for better breeding follows precise temporal patterns: during the dry season (in particular March-June) livestock moves from the North to the South to graze around sources of water, whereas during the rainy season transhumant livestock moves back to the North to utilize temporary surface water and rainy season grasslands [13–15].

These movements, potentially international, may cover long distances. Sometimes the interaction between nomadic/transhumant livestock herders on one side, and crop farmers on the other side, can result in conflicts. To maintain a peaceful cohabitation, member countries of the Economic Community of West African States (ECOWAS) have agreed to regulate livestock cross-border movements through (i) the delivery of International Transhumance Certificates (ITC), (ii) the creation of entry points and (iii) the recognition of transhumance corridors [10, 14, 16]. In addition, bilateral agreements between Mauritania and neighboring countries regulate international transhumant movements.

Aside from these transhumance movements, livestock are moved also over shorter distances to sell them in markets in order for the farmers to cover familial needs, in particular cereals not procurable in the arid areas [12], or at the occurrence of religious festivities like Eid al-Adha, the so-called Tabaski in Sahelian Africa. Tabaski is a major Muslim feast during which a young male sheep is traditionally slaughtered in many families. The Tabaski date is defined according to the lunar calendar and therefore it changes every year with respect to the Gregorian calendar (i.e. it advances by 13 days each year). In 2014 this event took place at the beginning of October and animals were moved during the previous month to provision markets and cities.

In addition, livestock traders collect animals in farms and later sell them at big markets. From there they are sent to major cities, most often on the coasts, to cover the ever-growing red-meat demand. Nowadays, a varying proportion of these commercial (and sometimes transhumance) movements is made by truck. Consequently, animals reared in northern arid areas can reach large urban markets such as those of Dakar, Abidjan and Lagos within two or three days.

However, little is known of the livestock mobility patterns and most of the scarce information is stored at a local level [14, 15], thus hindering the comprehension of mobility at a larger scale.

The mobility of farmers and their herds has the double potential effect of exposing healthy animals to new viruses upon their arrival or introducing infected animals into disease-free areas [13, 17], thus putting at risk origin and destination countries. Rift Valley Fever (RVF), Peste des Petits Ruminants (PPR), Foot-and-Mouth disease (FMD), Contagious Bovine Pleuropneumonia (CBPP) and Crimean-Congo hemorrhagic fever (CCHF) are important examples of transboundary diseases of ruminants in the region. Moreover, slaughtering and butchering methods that don’t strictly follow hygienic measures may result in pathogen transmission to humans [13, 17].

The occurrence of different types of movements (transhumance, trade) and the species’ own mobility patterns (e.g. specialized livestock markets for cattle, small ruminants…), result in complex mobility networks requiring intensive data collection campaigns to be deeply understood. The ITCs provide some information that can be used to quantify cross-border movements, such as origin and destination, herd size, frequency and vaccination status. However, due to different constraints (taxes, poor level of organization, ill-regulation), illegal movements still represent a large fraction of international movements [13]. At a national level, identification and traceability of livestock movements, as requested by OIE and World Trade Organization [18, 19], has not been fully implemented yet. In fact, the inaccessibility of some of the pastoral zones (conflict areas), the herders’ reticence to answer questions as well as the lack of telecommunication infrastructure to collect data, hamper initiatives to centralize mobility information.

In some cases, like in Senegal and Mauritania, some information about livestock movements is collected i) at veterinary posts (ITC and animal health certificates) and ii) at markets (market ledgers). Moreover, village leaders and herders can provide general accounts of livestock mobility, preferred routes, herd’s sizes and activity periods along the year. To collect these data, ad-hoc activities by veterinary services should be put in place. Up to now, only few of this information is available to researchers and animal health managers. However, compared to a situation of absence of data, this kind of information, even if imperfect, can be of uttermost utility to better understand mobility patterns in the region.

In this article, we present a descriptive analysis of livestock movement patterns in Mauritania during 2014, using data collected by the Centre National d’Elevage et de Recherches Vétérinaires (CNERV). We restricted the analysis to the description of mobility patterns in terms of involved species, transportation means, international vs. national movements and the occurrence of religious festivity in the period. We did not consider intercontinental trade, a small but growing market, due to the lack of information. Data were collected using low-budget and low-technology surveys in a short period of time, nevertheless, the dataset was rich enough to draw first conclusions on animal mobility in this area.

## Material and methods

Mauritania is located between the 15^*th*^ and 27^*th*^ degree North latitude and the 5^*th*^ and 17^*th*^ degree West longitude, delimited on the west by the Atlantic Ocean and confining with Morocco (North West), Algeria (North), Mali (East and South) and Senegal (South). The great extent of the territory in latitude explains the diversity of the climates encountered. The northern part, all along the border with Morocco and Mali, is hyper-arid [20], while the rest is arid, with some isolated areas of semi arid climate at the border with Senegal and Mali as shown in Fig A in S1 Text. Rainfalls are concentrated during the short rainy season from July to September, mainly in the South, as shown in Fig A in S1 Text. The annual rainfall ranges from 10 to 40 cm, with a rainfall peak generally observed in August [21]. The human population of Mauritania, which is around five million according to the official statistics, is mostly concentrated in a few cities in the southern part and on the coast. The animal population (cattle, dromedaries and small ruminants) is around four times as much as the human one, mostly concentrated in the arid zone of the South between the border with Senegal and the 17^*th*^ degree North latitude, Fig A in S1 Text. Mauritania’s economy is mainly based on livestock farming with almost 70% of agricultural GDP and 14% of the national GDP, provided by livestock-related activities [10, 12, 22]. International livestock movements are mostly concentrated at the border area with Mali and Senegal where it prevalently consists in cattle and small ruminants, while dromedaries are mostly traded towards Northern Africa [14, 23, 24].

In June 2015, an origin/destination survey was conducted by CNERV scientists in 12 out of the 13 Wilayas—a Mauritanian administrative unit corresponding to level one in the nomenclature of territorial units for statistics (NUTS1). The aim of the survey was to get an overview of 2014’s livestock movements and map the livestock movements. Two types of fonts were uses: data from livestock movement’s certificates stored at veterinarian posts; answers to ad-hoc surveys in villages. Data were collected from veterinary posts (VP) officers and village leaders (or an acknowledged individual when unavailable) through face-to-face or, in some VP cases, phone interviews. All the respondents (village leaders, officers..) involved in the field surveys were provided with detailed information on the goals of the study and the use of the data, also in the case of the estimations of illegal movements. The collection of movements certificates is a routine activity for VP officers. VP officers were informed of the activity and attended a preparation workshop. VP officers were asked to provide either aggregated information from the certificates either the certificates’ copies. Villages respondents,leaders or well-renown persons, were asked orally if they wanted to provide some information. The consent was given orally to overcome problems related to illiteracy. They freely decided to participate—or not, in the survey. In the positive case the interview was done at the moment and only once. In the case villages’ interviewees were not interested, investigators didn’t ask any question. As from the protocol, none of the respondents was interviewed more than once. The procedure, the survey protocol and data management have been approved by a committee formed by directors of CNERV (now ONARDEL) and of the National Veterinarian Service. The latter is the competent body for animal health in Mauritania. No identifying information of the veterinarian officers, herders, village leaders, investigators were recorded.

In accordance to the existing regulation, an animal-health certificate, or a transhumance one, must be delivered by the VP anytime a herd larger than 50 cattle or dromedaries, or 200 small ruminants (sheep and goats), moves towards markets or farms located outside the Wilaya. Furthermore, for transhumance movements, at least 2 herdsmen are supposed to accompany herds to obtain a certificate. One copy of each certificate is sent to the CNERV Wilaya delegation, one accompanies the herder along the way and a third one is left at the VP. The following information are reported: the origin, the destination, the starting date, the reason of the movement (market, farm, slaughterhouse, other), the species, the herd size and the transportation means (by foot or by truck). To complete the study with short-range movements and/or movements involving only a few animals, surveys were also done in villages. The village leader, or a well-renown person, was interviewed to provide estimates of the number of animals, their movements, the presence and the capacity of markets in the area and the existence of passage points for transhumance. Herd-sizes estimates were checked with local veterinary officers. In case of discrepancies between officers’ and respondents’ estimates, the former was retained. The coordinates of the villages were recorded using global positioning system (GPS) devices. A copy of the survey form is shown in Fig B in S1 Text.

**Fig 1:**
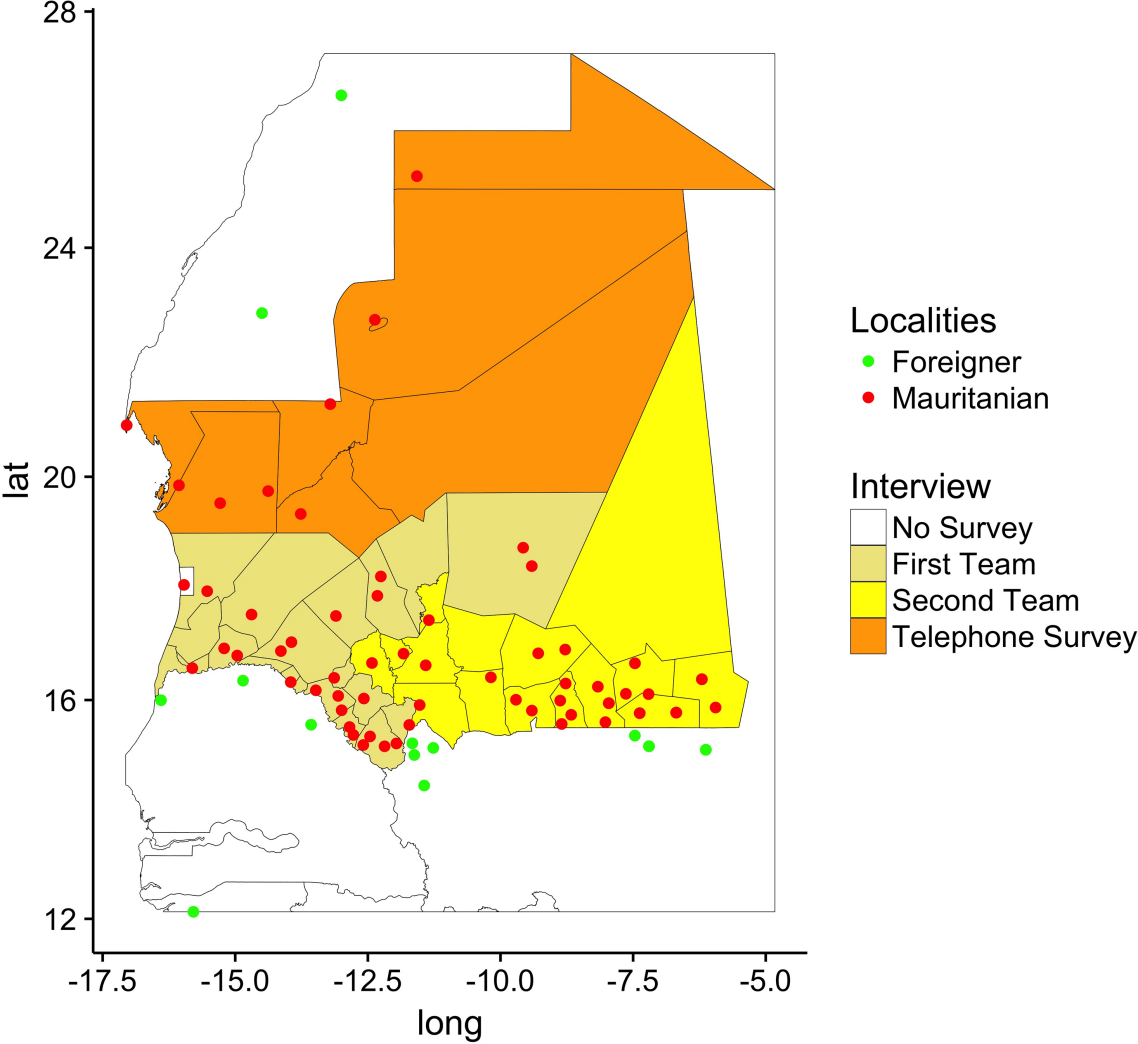
Survey design and locations. Mauritanian and neighboring-country locations are indicated by red and green dots, respectively. Wilaya colors refer to the interview type: (i) face-to-face surveys in Trarza, Brakna, Gorgol, Guidimaka and Tagant (first team, light brown); Hodh Ech Chargui, Hodh El Gharbi and Assaba (second team, yellow); (ii) phone surveys: Adrar, Inchiri, Tiris, Zemmour and Nouadhibou (orange). Map done using R version 3.3.1 (https://www.r-project.org).

In the Wilayas of Adrar, Inchiri, Tiris Zemmour and Nouadhibou only veterinarian officers at the VPs were interviewed, and only by phone call at their posts (Fig 1). These officers provided aggregated information from certificates collected at their posts. This information was recorded on an Excel worksheet. Due to some technical difficulties, no village interviews were conducted. Two teams were dispatched to the other Wilayas: the first to those of Trarza, Brakna, Gorgol, Guidimaka and of Tagant; the second to Hodh Ech Chargui, Hodh El Gharbi and Assaba. In this case, team members summarized the data from the hard copies of 2014’s certificates and stored on an Excel worksheet. At the same time, team members interviewed village leaders of the villages they met.

Moreover, face-to-face interviews were conducted at the Mauritanian transit points covering Podor and Matam (Senegal), Kayes and Nara (Mali), and Dakhla and Tichla (Morocco) to account for international movements towards neighboring countries. In the latter case, borders are closed for animal trade and consequently herdsmen let animals (mainly dromedaries) free to roam across borders till they are collected by partner herdsmen in the other country. Interviews with herdsmen in Dakhla and Tichla provided some first estimates of illegal movements between Morocco and Mauritania. Fig 1 shows the set of surveyed Wilayas together with the locations involved in livestock movements. The type of survey (phone call or face-to-face meeting) is indicated by the Wilaya color. The Mauritanian or foreign location of the points is coded by its color. Data collection took 2 months and they were all stored in Microsoft Excel worksheets. They were cleaned to discard duplicates in location name. When needed, their position and name were cross-checked on Google Maps and other software. For all the villages whose position we couldn’t determine exactly we used the centroid’s coordinates of the communes they belong to.

Cleaned data were used to build the Mauritanian livestock mobility network, with nodes corresponding to the villages at the origin or the destination of the movements. Movements originating from the same village and ending at the same destination were aggregated. A direct link was drawn between an origin-destination pair if at least a single animal moved from one of these places to the other. A link was characterized by the species of the animals (goats and sheep grouped together as small ruminants, cattle and dromedaries), the herd size (capacity), the month of occurrence, the transportation mean, the expected purpose of the movement (the main type of activities at origin and destination) and the national / international nature of the movement. A movement was considered as national if both origin and destination were in Mauritania, international otherwise.

We considered a one-month time step and built a monthly representation of the network (snapshot) with the corresponding number of nodes and links, and their features [5, 25]. A link was said to be active during a specific month if it appeared in the corresponding network representation. We used the terms “frequency” and “volume” to indicate respectively the number of times a movement with a specific set of features appeared in the database and the associated number of animals involved. Following network terminology [26, 27] we indicated with in- and out-degrees (in- and out-weights) the number (volume) of the incoming and outgoing movements to/from a specific location. We studied the degree and links’ volume distributions to identify possible hubs and preferential axes of movements. To provide some hints of the role of the networks on the diffusion of livestock diseases, we studied the connectedness properties of the network and the presence of strong and weakly connected components. Connectedness indicates the property of the network of being considered as a single graph (connected) or not (disconnected). Since we are dealing with a directed network, certain nodes can be reached only following specific paths. The strong and weakly connected components indicate the subsets of nodes that can be reached by all the other nodes in the subset (strong) or only by a fraction of them (weakly). These measures give some limits on the extent of locations that can be attained during an epidemics outbreak [28].

The network approach allowed us to map movement axes, identify possible differences in time, and specific species patterns. We used nodal region methods [29, 30] to highlight hierarchies in the network [31, 32] and the functional relations of the hierarchy. These methods rely on the definition of dominant flows and the results consist in the decomposition of the network in a set of smaller sub-networks formed by dominant links from peripheral nodes to central ones. In a directed network, a link between two nodes *i* and *j* is considered dominant if the largest fraction of outgoing flows from *i* is towards *j*, and the sum of incoming flows of *j* is larger than that of *i*’s. We selected outgoing links whose capacities represented more than 20% of the outgoing volume of the origin. This choice reduces the network to a set of 32 locations connected by 31 links. Despite the reduction in terms of localities and connections, the remaining network accounts for around 53% of the animal volume.The resulting partition consisted in smaller networks around dominant nodes (those towards which only dominant flows were directed) and corresponded to areas whose activities, in these case livestocks movements, are organized around a “central location”. In our case these centers correspond to markets and/or farms ruling livestock exchanges, and the sub-dominant locations represent the extension of their influence areas.

As stated above, data collected provided a photograph for each month of mobility in Mauritania. Some of the motifs found within one month can disappear during the following one, while certain axes of mobility remain present throughout the whole year. To describe the temporal evolution of the network and find constant structures in time, we classified the links according to the frequency of their activity. For each link, we counted the number of active months during a year. We then classified the links belonging to 4 different non-overlapping networks: backbone (present throughout the year), frequent (active for 7-11 months), intermediate (active for 3-6 months) and occasional (active 1-2 months). The correlation among pairs of networks was evaluated using the product-moment correlation method. To assess if the correlation was significant, we considered 1000 node label permutations of the networks and evaluated the product-moment correlation. An experimental p-value was estimated as the fraction of correlations whose values were higher than the estimated one (Quadratic Assignment Procedure) [33].

To assess the variation of the network along the year we use the Jaccard index *J*: this is a measure used to quantify the difference between two sets *A* and *B* as follows

$$\begin{array}{c} J=\frac{dim(A\cap B)}{dim(A\cup B)} \end{array}$$ (1)

Two sets that are identical have a Jaccard index close to one, vice-versa dissimilar sets have a Jaccard index close to zero In our case *A* and *B* could be either the set of nodes or links of the networks in 2 consecutive months [1]. For weighted networks we can use the Jaccard index *J*′:

$$\begin{array}{c} J^{\prime }=\frac{\sum \text{min}.\text{weight}(A\cap B)}{\sum \text{max}.\text{weight}(A\cup B)} \end{array}$$ (2)

where min.weight and max.weight indicate the minimum and the maximum weight of a link. Two sets are increasingly similar the more *J*′ is close to 1 [34].

We used Jaccard index *J*′ to identify important “periods” for the mobility network evolution. Each monthly network has been considered as a snapshot of an evolving network. We can suppose that if some dynamics are undergoing the network evolution, the snapshots could follow a trend in their properties, and with them, their Jaccard indexes. Periods correspond to intervals of time during which a specific network dynamic is taking place. Snapshots belonging to different periods are, in principle, different in terms of structural properties. As a consequence, the behaviour of similarity measures, like Jaccard indexes, follow specific trends and turning points, corresponding to months where the trends abruptly change, representing changes in periods. To unfold the dynamics that underlie network evolution and identify the above-mentioned turning points, we followed the procedure by Berlingerio et al. [34]. This method aims at regrouping consecutive snapshots in clusters based on a “dissimilarity function” on the Jaccard indexes of the snapshots: members of the same clusters are more similar to each other than they are with the others, thus constituting a period for the network.

We used R [35], a language and environment for statistical computing, as well as add-on packages (ggplot,flows,network) [36, 37] to perform data analysis and plotting.

## Results

### Descriptive analysis

**Fig 2:**
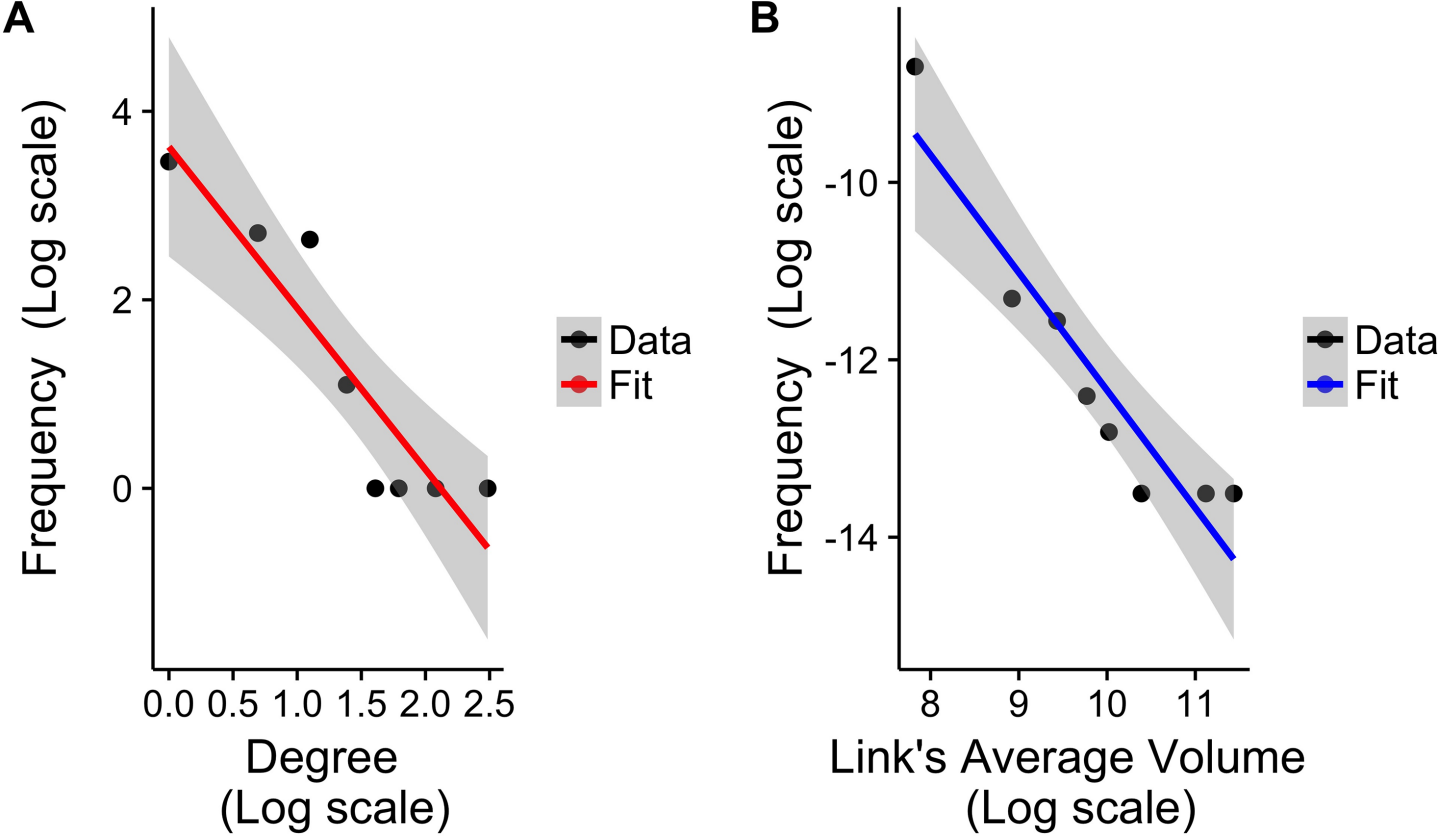
Distributions of the degree and average link’s volume. (A) degree distribution for the aggregated network: the network follows a power law distribution *P*(*k*) ≈ *k*^−*γ*^, *γ* = −1.71 (B) Average link’s volume distribution: the network follows a power law distribution *P*(*w*) ≈ *w*^−*η*^, *η* = −1.33.

The database contains information on movements from January to December 2014. A total of 2,259 movements between 87 different locations (Fig 1), involving 7.2 million sheep and goats (small ruminants), cattle and dromedaries were recorded. In general, the network aggregated over the entire year (from now on “Overall network”) follows a power law for the degree distribution, with node connections ranging from 1 to 12, and a power law for the link’s average volume distribution, with volumes ranging from 40 to 91000, as shown in Fig 2. The network is connected, with a low clustering coefficient (around 1.2%) and a small diameter (7 links). All these facts indicate the presence of hubs (Kiffa, Aleg and Maghtaa Lahjar) and major exchange routes (in particular the axes Adel Bagrou-Nara and Rkiz-Podor). However, some of these characteristics and the role of nodes could change along the year and depending on the species involved. Additional information is available in Table A in S1 Text.

Most of the locations were in Mauritania (73 / 87) and concentrated in the southern part of the country where animal densities are the highest. International locations were in Senegal, Mali,Guinea, Guinea Bissau and Morocco. International and national movements’ frequencies were similar. However, international movements accounted for 70% of the volume. Only 9% of the accounted animals were imported (mostly small ruminants), thus making the country an active livestock exporter. Less than 18% of the animals were transported by truck: the most common mode was by foot, including international movements (94%). When considering volume, the largest part also walked to the destination (91% of the annual volume). At the national level, the two transportation modes were almost equal with a slight preference for walking both in frequency (55%) and volume (63%). More than half of movements involved small ruminants (two thirds of the annual traded volume), followed by cattle (26% of the movements and 30% of the volume) and dromedaries (17% of the movements and 8% of the volume). National movements were slightly more frequent for small ruminants (53%) and dromedaries (59%). Cattle movements were equally distributed between national and international. In volume, 47%, 70% and 80% of the dromedaries, small ruminants and cattle were exported.

As stated above, health certificates don’t collect information about single production units but in most of the cases herdsmen are responsible for composite herds of different owners. In the following paragraphs we use the original term *zone d’elevage*, as in the certificates, to indicate any location where livestock is raised. This could correspond to actual farms but also pastoral zones and entire villages where animals are herded together [38, 39]. All movements’ origins were *zones d’elevage* except for two market places. The situation was different for the movements’ destinations. Among the 38 *zones d’elevage* locations, nine were also market places, three were also slaughterhouses and one was also a resting area. Apart from the *zones d’elevage*, there were 12 other market places.

Table 1 shows the distribution of movements according to the origin and destination activities and the corresponding volumes (given as a percentage of the total number of exchanged animals). The vast majority of movements (93%) occurred from *zone d’elevage* to markets or to other *grazing* areas for pastoral activities and fostering practice (“confiage”) [38–40]. The majority of the commercial movements were to national markets (57%), whilst movements between *zones d’elevage* were mostly international (57%). Other movements originating from farms accounted for less than 2% of the movements. The remaining 5% of the movements corresponded to movements between Mauritanian markets or from markets to *zones d’ elevage*. In volume, however, almost three quarters occurred from *zone d’elevage* to *zone d’elevage*, 79% of them outside Mauritania. Only a quarter was sent from *zones d’elevage* to markets, equally distributed between Mauritanian and foreign ones.

**Table 1:** Distribution of movements and volume (as a percentage of the total volume) according to the type of activity at the origin and destination.

Destination					
		Slaughterhouse	Zone d’elevage	Market	Resting Area
		Movements (Volume %)			
Origin	Zone d’elevage	51 (0.11)	1072 (73)	1136 (25)	4 (0.03)
	Market	0	18 (0.4)	104 (1.4)	0

**Fig 3:**
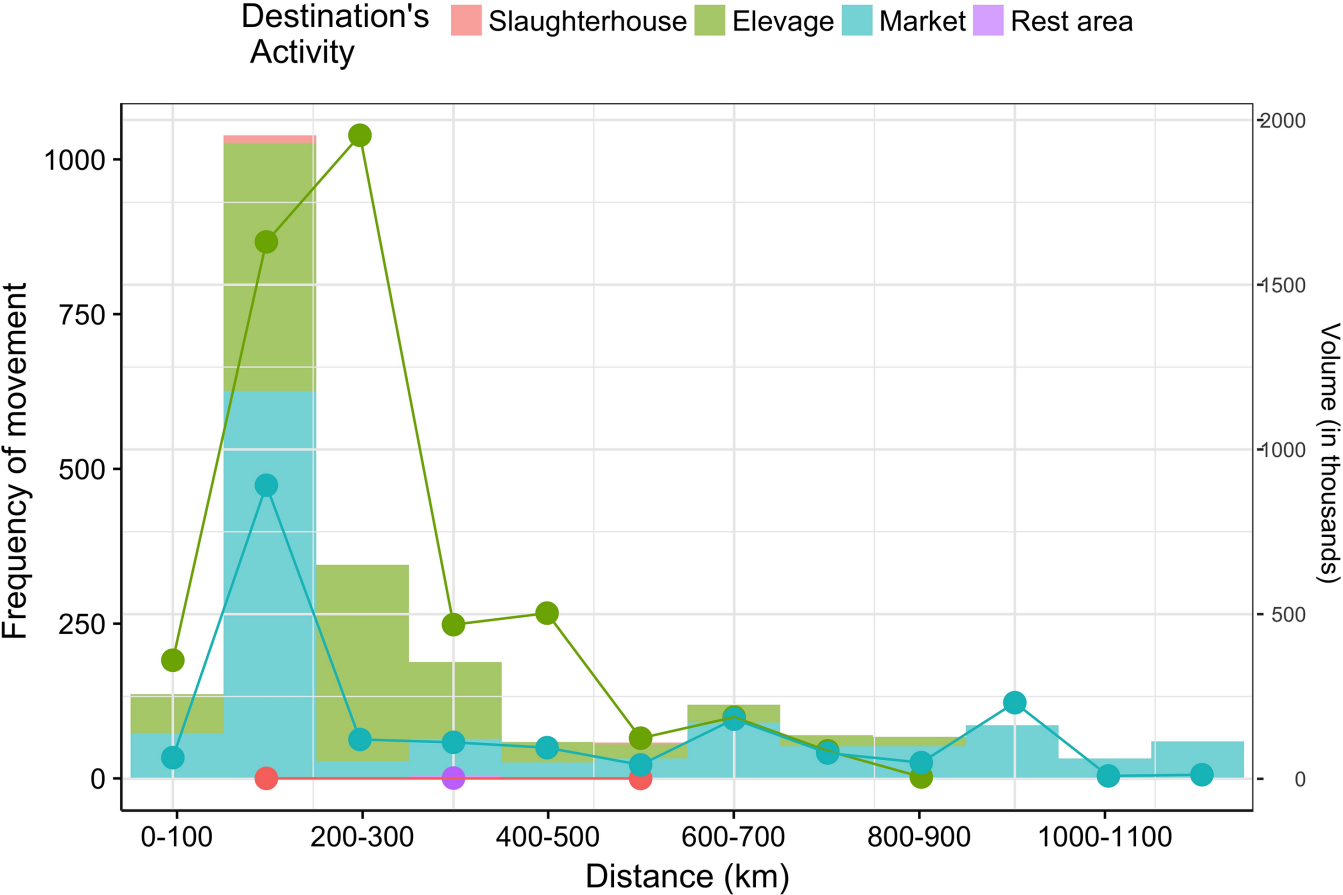
Distance distribution by type of destination activity. Histogram corresponds to the number of movements among locations at a certain distance, solid lines to volume exchanged by locations at a certain distance. Color is related to the destination activity type.

Fig 3 shows the distribution of distances and the corresponding volumes originating from *zones d’elevage*. Most movements occurred within a short range, between 100 and 300 km. There were fewer movements above 300 km. In volume, most between-*zones d’elevage* movements did not exceed 300 km and *zone d’elevage*-to-market movements were even shorter (maximum distance of 200 km). Beyond these limits, the number and volume of movements decreased, indicating that longer movements involved small flocks. This can be a consequence of the fact that most of the flocks are moved by foot: large flocks can be moved in a cheap and convenient way, but on a shorter distance, as shown in Fig D in S1 Text.

**Fig 4:**
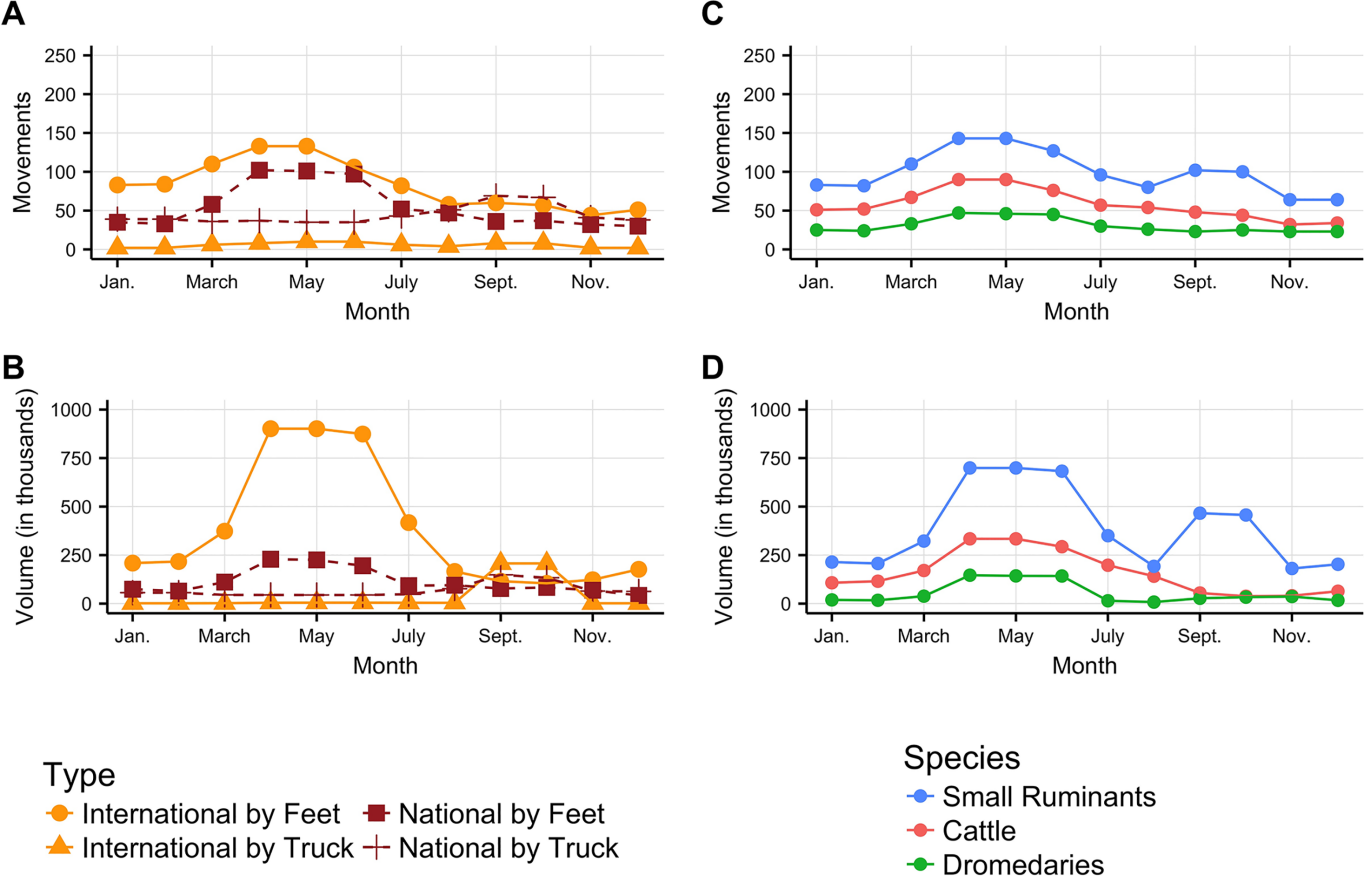
Number (top) and volume (bottom) of movements along the year by features (column): Type of movement (A,B), species (C,D).

Frequency and volume changed during the year. Fig 4 shows their temporal patterns according to the movement type (national vs. international, transportation mode) and species. Between January and August, the majority of movements were done walking, on national (31%) and international paths (48%) (Fig 4A). Trucks were rather used at the national (17.5%) than at the international level (3.5%). Most animals walked, either on international (75%) or national routes (18%). Volumes moved by truck were smaller at the national (6.5%) and negligible (0.5%) at the international level (Fig 4B). Conversely, in September-October (Fig 4B), most animals were moved by truck for international (38.5% of the volume) or national (26%) movements. Those who walked were equally distributed among international (20%) and national movements (15.5%). Species movements had different temporal patterns. Small ruminants were the most traded animals in frequency (Fig 4C) and volume (Fig 4D). Compared to dromedaries and cattle, whose trade dropped off after the summer, small ruminants movements showed a surge in September-October, related to the Tabaski during which millions of sheep are slaughtered on the same day.

**Fig 5:**
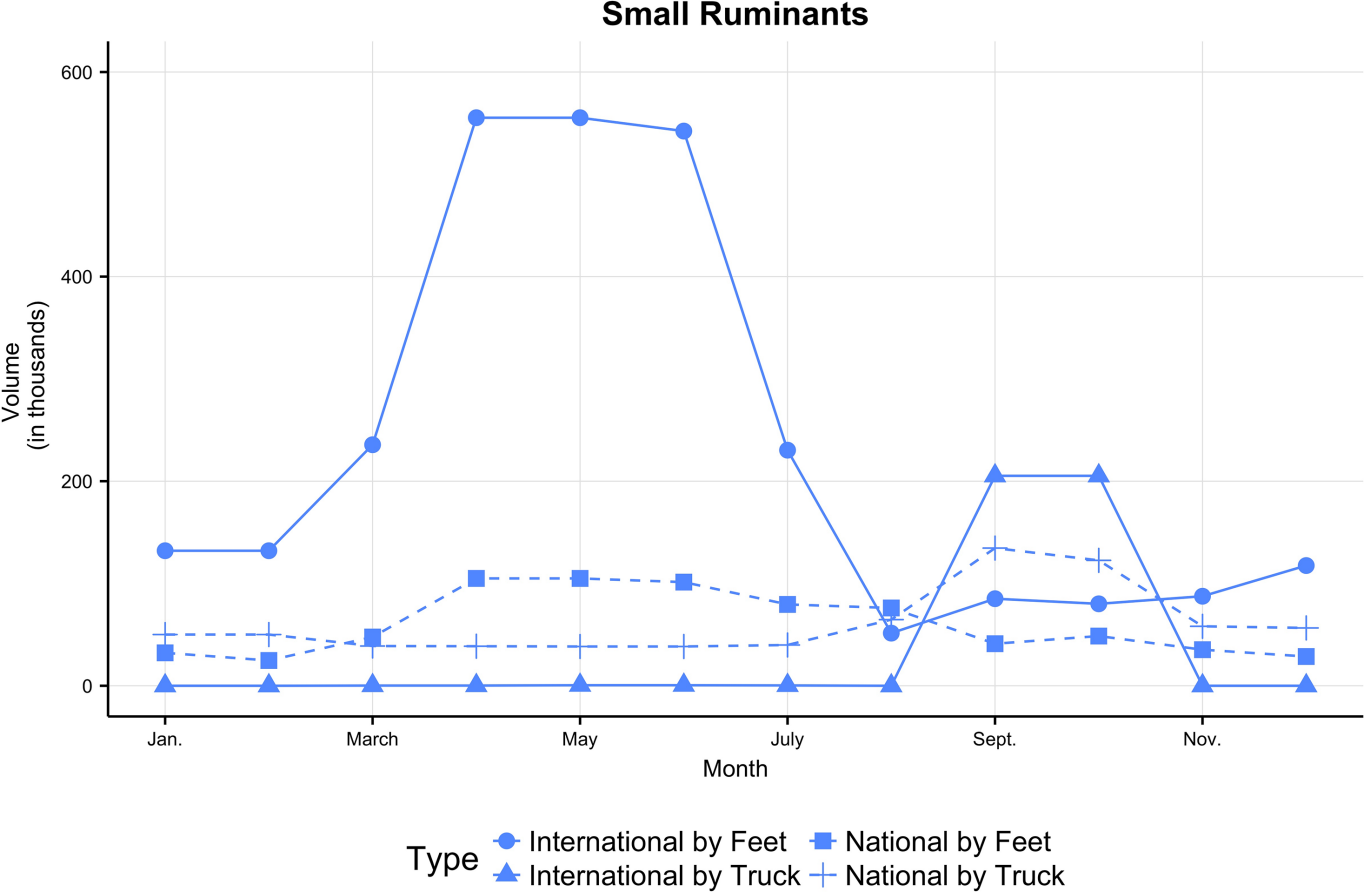
International and national monthly volume of small ruminants moved by truck or on foot. The solid lines correspond to international movements, the dashed one to national movements. The points shapes correspond to movement by truck (triangle and cross) or foot (circle and square).

Tabaski-related trade affects mobility patterns in other ways. In Fig 5 we focused on small ruminants movements that constitute the largest part of the dataset. As seen above, walking was the most common transportation mode for small ruminants in the first part of the year (Fig 5). However there was an inversion before Tabaski when transportation on trucks for small ruminants became the dominant way. This abrupt change in pattern was dictated by the sharp increase of sheep demand on urban markets. Moreover, the national volume of ruminants moved by foot was nearly constant throughout the whole year. A comparison between the species involved is shown in Fig C S1 Text.

### Spatial and temporal analysis

**Fig 6:**
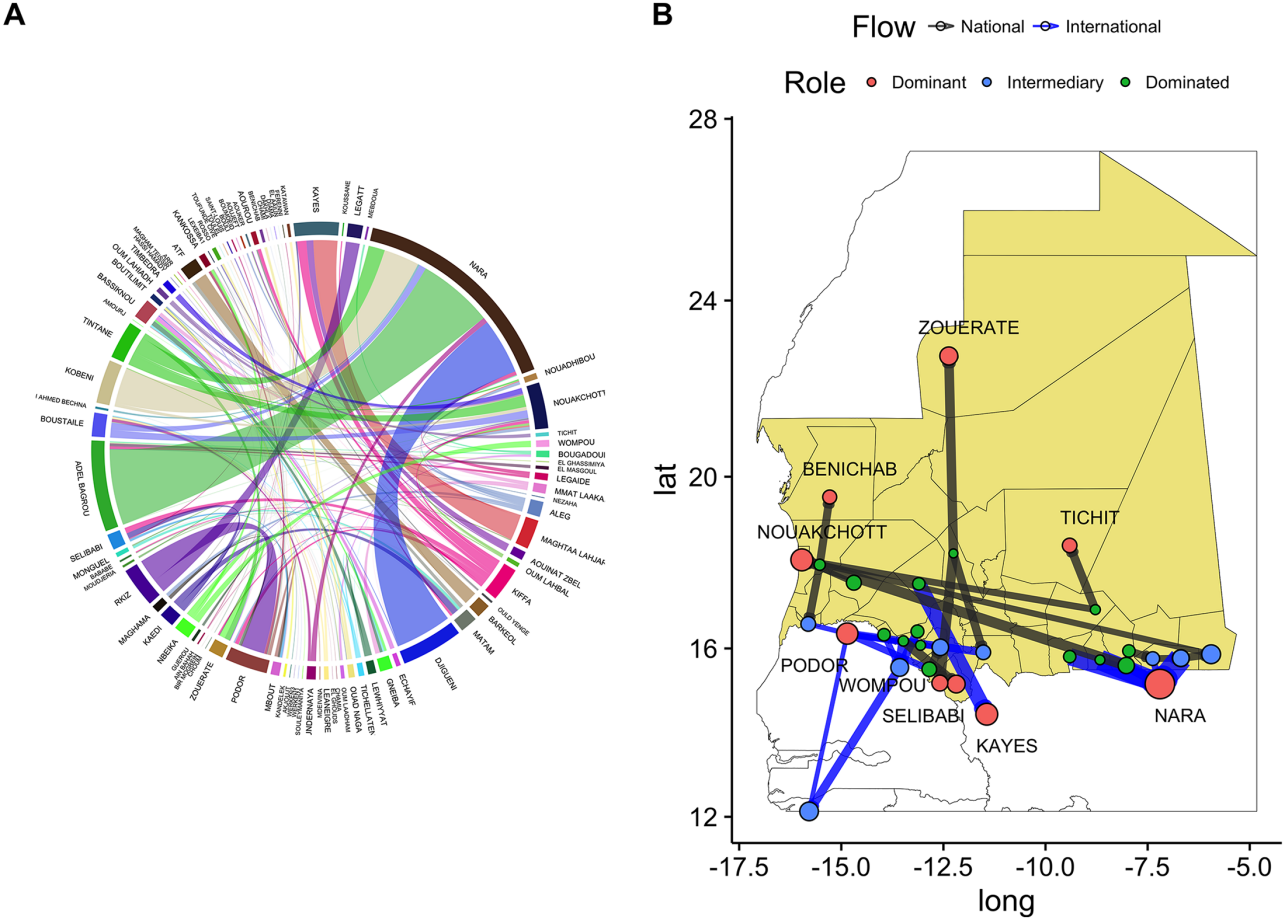
Mobility network. A) Chord Diagram for the static representation of the network. The size of the sector is proportional to the volume towards the location, color of the band indicates the origin of the flux B) Dominant flows of animals national (black) and international (blue). Map done using R version 3.3.1 (https://www.r-project.org).

A chord diagram of the animal flows is shown in Fig 6A together with a map of the principal axes of movement, Fig 6B.

In Fig 6A the size of the sector is proportional to the animal inflow and the size of the connecting band to the volume of animals exported. As can be noticed, Nara, Adel Bagrou, Podor, Djugeni, Kobeni and Nouakchott play an important role. We also notice that the flows from all these localities, except for Nouakchott, are towards Nara (Mali). Nouackhott, on the other hand, depends from a group of smaller locations for its inflows. Other reported locations correspond to intermediate stop-overs on the roads to Mali and Senegal. From there, animals were loaded on trucks to reach larger markets like Nouakchott, Zouerate, Kiffa and Nouadhibou which are also the most populated areas. At the national level, we identified two main axes (Fig 6B in black): (i) from the South-East regions of Mauritania to the coast; (ii) from the South to Zouerate in the North. At an international level we notice that most of the dominant flows are concentrated along the southern border, in 2 distinct groups (towards Senegal and Mali respectively) with a specific flow towards Kayes. We notice also that Nara, Podor Kayes and Wompou (international locations) are also dominant. Finally, the majority of the intermediary location are at the interface between national and international dominant flows, fusing together the movements along two main international axes: from the North of Mauritania to Senegal and Guinea Bissau; from Nara (Mali) to Nouakchott (East-West).

**Fig 7:**
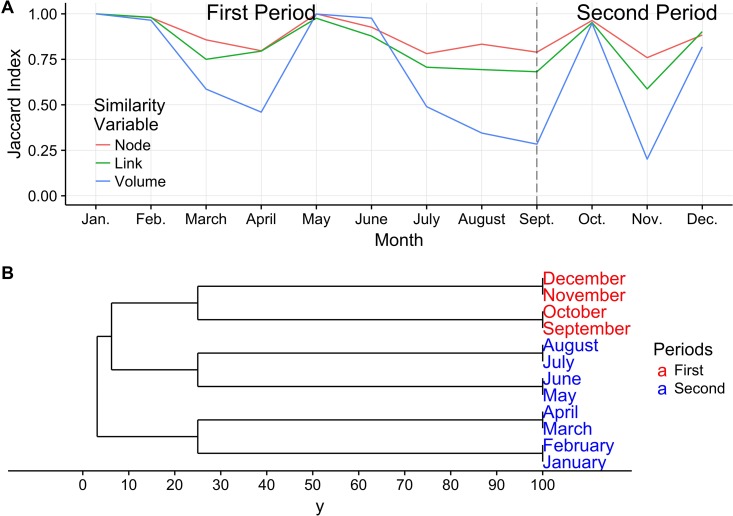
Network variation along the year. A) Temporal variation for the Jaccard indexes of nodes, links and links’ volume B) Identification of mobility periods.

A static representation of the mobility network fails to capture some peculiarities of the temporal patterns. Depending on the time of the year, the distribution of links can change and with it some properties of the network, whilst other can remain throughout the entire year. Fig 7A shows the temporal behavior for the Jaccard indexes, for nodes, links and link’s volume throughout the year. Variations in the Jaccard indexes for nodes are limited over the year, indicating a certain stability in terms of structure of the network, related to its finite size. The three indexes are almost equal to one around May and June, the period of the peak in movements, and in October, the Tabaski period, indicating a sort of regularity of the network in these periods. Nevertheless, the Jaccard index *J*′, relative to the link’s weights, is widely fluctuating along the year. In particular is sky-rocketing between August September and October passing from around 0.3 to almost 1. This indicates that despite some small changes in the network between August and September, the volume of animal exchanges has varied a lot due to the fact that ruminant transhumance almost came to a hault and to the upcoming arrival of Tabaski. The procedure form Berlingerio et al. [34] divides the year in 2 periods, Fig 7B: January-August mostly related to routine movements; and September-December mostly related to Tabaski-related trade. As we can notice, the partition month corresponds to the month of higher variation of the Jaccard index for the links’ weight

**Fig 8:**
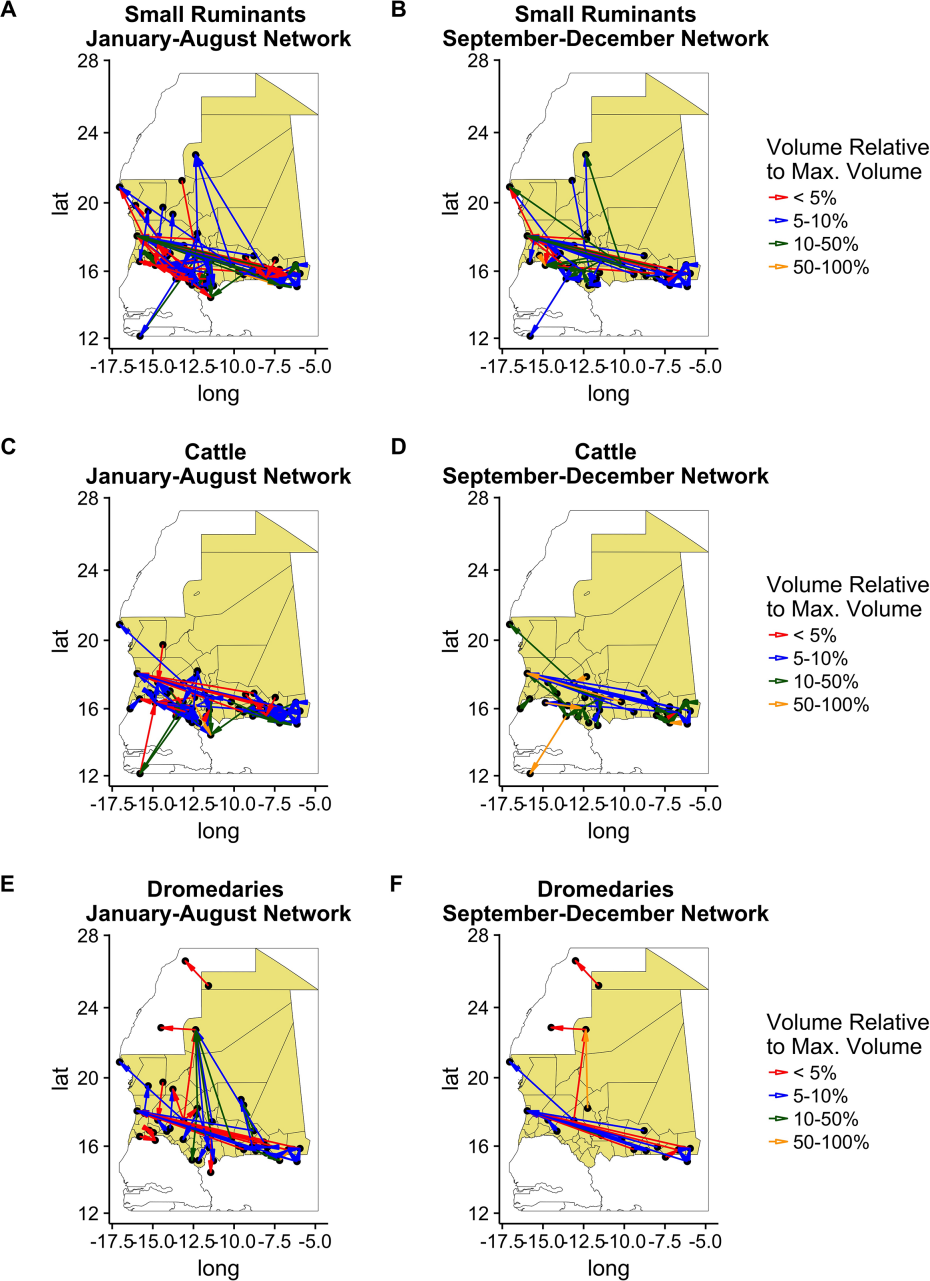
Mobility network along the year according to the species. Each line corresponds to the network specific to a species. Left column, network during the first period. Right column, network around Tabaski period. Color indicates the volume moved along the connection, normalized to the maximum volume for the period of the year. Maps done using R version 3.3.1 (https://www.r-project.org).

Fig 8 shows the mobility patterns during the two periods of the year, for each species.Superposing the different networks, we have a snapshot of the networks as shown in Fig E S1 Text.

In both periods, movements were concentrated in southern Mauritania, with a few long-range movements from the south to the coastal cities and the North.

Throughout the year, small ruminant networks stretch from northern Mauritania to Guinea Bissau (Fig 8A and 8B). However, in the second period, the network had fewer nodes and links, and movements to coastal cities became more important. Cattle movements were mostly concentrated in the southern and greener part of Mauritania (Fig 8C and 8D) with a few movements to Nouadhibou. Finally, only dromedary movements were recorded to Morocco (Fig 8E and 8F). The axis Nbeika-Zouerate became even more important during the second semester.

The Table 2 compares characteristic of the different species’ networks between the two period and the aggregated network. We notice that between the two period there is a reduction in number of links and nodes involved in the movement of the species, except for bovine movements that involves almost the same amount of locations. As we can see, except for the bovine network (overall and during the first period), all the networks are disconnected. The size of the weakly connected component gives an estimate of the size of the largest component of the network. Consequently, although the network is disconnected, a giant sub-network appears, almost the size of the network, and just few nodes are isolated

**Table 2:** Summary of network characteristics by species and time of the year. Nodes and links are counted during the specific period or through all the year (ll over the year). Connected indicates if the network can be considered as single entity (Yes) or not (Not). Weak and strong indicate the sizes of the weakly and strongly connected component.

Species	Period	Nodes	Links	Connectedness	Strong	Weak
All	Overall	87	147	Not	10	83
	January-August	85	131	Not	4	83
	September-December	63	89	Not	4	61
Small Ruminants	Overall	78	110	Not	5	76
	January-August	73	94	Not	2	66
	September-December	56	67	Not	3	52
Cattle	Overall	71	96	Yes	6	71
	January-August	71	93	Yes	1	71
	September-December	45	49	Not	3	39
Dromedaries	Overall	51	51	Not	1	35
	January-August	51	49	Not	1	31
	September-December	28	28	Not	1	26

**Fig 9:**
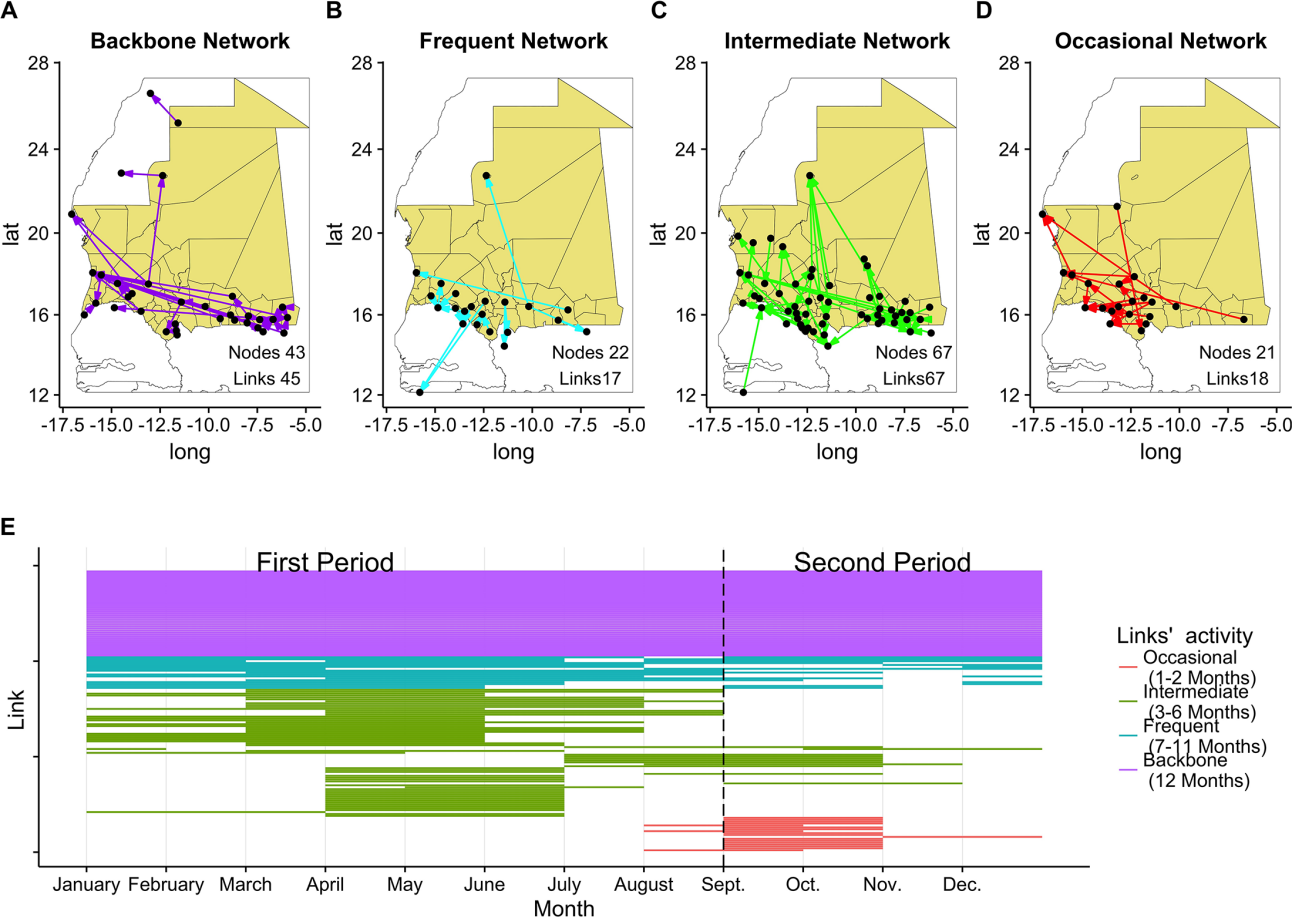
Geographical representation for the four networks. (A) Backbone, (B) Frequent, (C) Intermediate, (D) Occasional, and (E) Link activity along the year: each line corresponds to a link; a segment is drawn every month a link is active; colors correspond to the frequency they appear during the year, and consequently to the network they belong to. Maps done using R version 3.3.1 (https://www.r-project.org).

Fig 9A–9D show the geographical distribution of the 4 different networks defined above (Backbone, Frequent, Intermediate and Occasional). The backbone network (Fig 9A) presented long-range connections between northern and southern regions, as well as from the coast to the most inner areas. Short-range connections were present across the borders. Moreover, there was a constant trade flow between Mauritania and Morocco mostly related to dromedaries. Frequent network (Fig 9B) was mostly concentrated at the border with Senegal, with few long-range connections to Guinea Bissau and Zouerate in North Mauritania. Intermediate network (Fig 9C) was the largest regarding nodes and links, with two clusters at the borders with Senegal and Mali. The occasional network (Fig 9D) was very dense at the border with Senegal and exhibited a few long-range connections between south-western Mauritania and major cities on the coast. The 4 networks were characterized by the annual frequency of their links. A graph representation of the 4 networks is shown in Fig F S1 Text highlighting some of their topological features. All 4 networks were disconnected, presenting a large connected component made of at least 50% of the nodes. Moreover each network connects different sets of locations, as demonstrated by the low values of the Jaccard index for the nodes [0.19; 0.34]. Performing QAP test on the 4 networks has shown non-significative correlations among tests in S1 Text Table B. The 4 networks carry out different functions at different periods of the year and this could explain the reason they are almost uncorrelated.

Each line in Fig 9E represents the activity of a link: blank spots correspond to months when there was no exchange on that link; colored spots indicate that at least an animal was exchanged along the link. The color code is shared with Fig 9A–9D and indicates the annual activity frequency. The partition in two periods was based on the conclusion of the previous section and supported by applying the procedure of Berlingerio et al. [34].

Almost one third of the links were present throughout the year. Twelve per cent were active during one or two months per year and were also present during the second semester. The remaining links were active for a period ranging from 3 to 11 months, mostly during the first semester. Apart from the backbone network, active by definition throughout the whole year, different link classes had specific patterns: occasional links were active mostly during the Tabaski period; intermediate links were mostly present during the first semester; frequent links were equally distributed between the two periods.

Table 3 summarizes some features of the movements along the different networks and the aggregated one (Overall), during the two semesters: (i) network characteristics; (ii) the amount of animals exchanged; (iii) the specific composition; (iv) the transportation mode; (v) the fraction of national and import/export animals; (vi) the origin-destination types to account for the reason of the movement. Intermediate network accounted for almost half of total volume exchanged, whilst the occasional network only represented 3%. The backbone network and the frequent network accounted for 20% and 30% of the volume, respectively. In all the networks, small ruminants represented the dominant volume of traded animals. For all the networks, except the occasional one, the volume abruptly decreased during the second semester. In particular, for the intermediate network, the volume decreased almost fifteen times between the 2 periods.

**Table 3:** Characteristics of the four networks (Backbone, Frequent, Intermediate, Occasional) and the Overall one in the two periods of the year (January-August, September-December). Characteristics are summarized by species involved, means of transportation, national or not and reason of the movement (types of activity at origin and destination). Volume is expressed in million heads. Percentage referred to the volume specific of the period.

	Period of the year	Overall		Backbone		Frequent		Intermediate		Occasional	
		First	Second	First	Second	First	Second	First	Second	First	Second
Network	Nodes	85	63	43	43	22	21	67	17	5	21
	Links	131	89	45	45	17	15	66	11	3	18
	Volume (in millions)	5.6	1.6	1.0	0.5	1.5	0.7	3.1	0.2	0.01	0.2
Percentage(%)											
Species	Small ruminants	60	81	73	80	61	86	56	50	83	93
	Cattle	30	12	19	15	36	11	31	13	17	7
	Dromedaries	9	7	8	5	3	3	13	37	0	0
Transportation	Truck	7	50	38	54	3	53	0	6	0	75
	Foot	93	50	62	46	97	47	100	94	100	25
Movement	National	27	40	63	74	7	16	24	52	83	28
	Import	7	8	29	18	3	1	1	14	17	1
	Export	66	52	8	8	90	83	75	34	0	71
Origin-Destination	*zone d’elevage*-*zone d’elevage*	85	34	24	9	98	42	99	95	100	4
	*zone d’elevage*-Market	13	64	68	83	2	58	0	5	0	95
	Other	2	2	8	8	0	0	1	0	0	1

Not only the volume of traded livestock changes between period, but also their destinations. In fact, with the exception of occasional network, the percentage of animals exchanged at the national level increased between the first and the second part of the year. On the other hand, most animals exchanged on the occasional network were sent to foreign markets around the Tabaski period. The role of the frequent network changed between the two semesters: during the first one, it was a pastoral network connecting *zones d’elevage* and grazing areas; during the second semester, it was mainly used to deliver animals for consumption. Moreover, to provision cities in a short delay, most of the animals were transported by trucks, thus switching from the foot transportation of the first semester. The intermediate network was a special case: during the second semester, it was the only network connecting *zones d’elevage* among themselves rather than to the markets. Those movements were still done walking, thus indicating that this network was related to the background pastoral activities.

In short,as shown in the first column of Table 3, during the first period of the year (January-August), most network activities were devoted to international, between-*zones d’elevage* movements. During the second part of the year (September-December), the volume of exchanges decreased: the activity was mostly commercial, involving national (backbone and frequent networks) or international markets (occasional network). Pastoral activities were still present, at a finer spatial scale: as a matter of fact, the activity of the intermediate network was almost unchanged.

## Discussion

Mauritania is at the interface between northern and sub-Saharan Africa. The description of animal mobility gives a picture of the nature and the importance of social, economic and health features linking these regions. Thus, making livestock trade and transhumance safer is an important issue for Mauritania and neighboring countries. With this respect, a better knowledge and understanding of the livestock mobility patterns in this area could provide essential information to enhance the national animal diseases surveillance system and the development of control measures for animal and zoonotic diseases as well as to improve the efforts coordination at a regional level.

Previous studies on animal mobility in this region were either very general and qualitative [41], focused on specific areas [42] or restricted to specific movements—e.g. transhumance or trade, or animal categories—e.g. cattle or sheep [43]. Thus, we miss an overall, quantitative picture of the livestock mobility patterns and their drivers. In our case, the survey data were collected during a comprehensive activity encompassing most of the Mauritanian territory. Moreover, these survey data were cross-checked and validated with animal health certificates. Though the field work was fast, a large amount of time was needed to prepare it. Local authorities and partners used to work for almost a year to design the survey, organize the field work, capitalize pre-existing information, select villages to investigate, contact veterinary officers and train surveyors. Field survey data were completed with the animal health certificates and ad-hoc interviews provided a description of local movements (not recorded on the certificates). The volume of animals involved in local movements was confirmed by local veterinary officers who had a good knowledge of the area.

Our analysis has highlighted some characteristics of livestock mobility patterns in this area that should be taken in account when implementing surveillance activities and/or control measures:

- Between-*zones d’elevage*, transboundary exchanges are more important than commercial and national movements. Indeed, these international movements occur in the frame of regular pastoral activities in the area, like *Confiage*. This is a common practice among livestock-keepers to avoid disease outbreaks and climate shocks: part of livestock is sent to a fostering keeper in far regions till the end of the epidemic or drought. In most cases keepers are linked by parental ties [32, 38, 39]. Because of the large herd sizes and the high costs of truck transportation, walking was the preferred way of moving animals.
- Livestock mobility is a dynamic phenomenon whose properties change along the year depending on the species and the period of the year. In general, small ruminant movements stretched from northern Mauritania down to Mali (and then probably later to the Ivory Coast and the shores of the Gulf of Guinea), Senegal and Guinea-Bissau; cattle and dromedary movements were mostly concentrated in southern Mauritania, as well as from inner Mauritania to Nouakchott and Nouadhibou, with a few occurrences towards Guinea-Bissau for cattle, and substantial flows to southern Morocco for dromedaries.

Beyond these trends, important seasonal variations might impact the spread of epidemics. In small ruminants, a major exportation peak—involving circa two million heads, occurred between April and June (Fig 5), i.e., during the hot, dry season and the so-called “hunger gap”. Two main factors explain this peak:

- At this time of the year, temporary surface water has dried up and grasslands are exhausted or have lost their nutritional value. Consequently, farmers usually sell the sheep and goat offspring, most of them being three to six-months old: as a matter of fact, parturition peak ranges from November to February in small ruminants in this area [44–46]. Also, farmers need cash to cover household needs, particularly in cereals [47]. These offspring are either brought to the village markets for sale by the farmers themselves or purchased by specialized traders to be sold on the main small ruminant markets (e.g. Kaolack in Senegal). They are bought by smallholders for subsequent fattening using local agricultural by-products (groundnut hay, groundnut cake, cotton seed…). The main purpose of this commodity chain is to provide consumers with sheep for the Tabaski and other religious or familial festivals [43].
- Aside from utilizing the offspring, many farmers send their reproductive stock southwards, mostly in Senegal and Mali, where grasslands and water are more abundant. They stay there till the beginning of the next rainy season. However, these transhumance movements usually occur sooner than March: most often in October-November, i.e. just after the end of the rainy season [48].

A second, less marked peak was observed in September-October in small ruminants, involving more than 900,000 heads as a whole. This peak was clearly related to the Tabaski. It is worth noting that the estimated figures in this survey are comparable with those of the official agreement between the Senegalese and Mauritanian veterinary services: this agreement was signed between the two countries to avoid supply disruption on the Senegalese sheep markets with the consecutive abrupt rise in sheep prices and possible social troubles.

Tabaski is affecting the livestock mobility patterns in many ways, changing the role of the largest part of the mobility network, rerouting animals from export towards national markets, creating new movements (indicated in the paper as occasional network), increasing the fraction of animals moved by truck, and consequently moving faster, to provision major urban areas.

Understanding the mobility patterns in these two peak periods (April-June and September-October) can help planning surveillance and control measures against animal and zoonotic diseases. Aside from mobility data, other factors should be included (like rainfall, temperature, environmental conditions) that can affect the risk of transmission of certain diseases, in particular vector-borne infections like RVF or CCHF.

Between April and June, due to the large volume of exported livestock, the risk of introducing diseases in neighbor countries is higher: for instance, diseases occurring in Mauritania might easily spread to Mali and Senegal, and later spread to the whole region. Fortunately, this livestock-movement peak occurs during the hot and dry season when vector activity is at its lowest level. Therefore, directly or indirectly transmitted pathogens like FMD, PPR or CBPP should be targeted for increased surveillance and control at this period.

Heading towards Tabaski, despite the reduction in livestock movement compared to earlier periods, the risk of epidemics is increased by the rapidity of animal movement: most of the livestock is, in fact, moved to markets using trucks. Thus, an epidemic can spread over longer distances in shorter times. In the last years, Tabaski has been occurring in the middle of the rainy season, when the abundance of potential mosquito vectors (like and *Culex Poicilipes* and *Aedes Vexans*), are the highest along the Senegal River [49–51]. Because of this, the risk of triggering RVF epidemics is increased by the combined effect of the peak in livestock mobility, the rapidity of their movements and the higher vector abundances. This might well have happened in 2010, when an RVF outbreak occurred in northern Mauritania after exceptional rainfall: Mauritanian farmers brought their animals (small ruminants, cattle, dromedaries)—and most likely the RVF virus as well, from southern Mauritania by truck to benefit from the unexpected resources in surface water and grasslands [52, 53]. Antibodies against RVF virus were later found in Moroccan dromedaries [54], suggesting that the spread of this virus in Morocco had occurred via livestock trade, as it had been the case between the Horn of Africa and the Arabic peninsula in 2000 [55], or between the East African mainland and the Comoros Archipelago—and Madagascar in 2006-2008 [56, 57].

An option to consider when planning surveillance measures is to concentrate resources (staff, equipment) on known commercial or transhumance corridors during the above-mentioned periods (March-June, and one month before and after Tabaski) rather than all year long. This option could result in a higher chance of finding the diseases while reducing costs by shortening the periods of operation. Similarly, control measures such as vaccination could be implemented before March (PPR, CBPP) and/or a few months before Tabaski (RVF), to protect the targeted animal populations against these diseases and thus reduce the possibility of spread to other countries.

These data are far from perfect and in some Wilayas in the North, data are missing for practical constraints. However, animal and human populations are very small in these regions of Mauritania. Another weakness of these data is the lack of information on animal movements outside Mauritania: during the survey, data regarding international movements were collected on the country entry point and not further. Consequently, we do not know the final destination for the animals. Furthermore, a common practice in West African countries consists in grouping animals from villages for grazing and/or transhumance movements. Because of this, our data only captures movements of large (mostly composite) herds and movements of single owners cab not be part of them. Moreover, due to the restriction for the emission of ITC, small herds, if any, use informal frontier points during transhumance to avoid any administrative control. Further studies on transboundary movements were carried out by the partners after the end of this study, and results will be shortly available.

The year 2014 was one of the driest in the region in the last decade. Hence, water and forage resources were scarcer and animals had to move earlier than usual to undertake the transhumance. In 2014, Tabaski was at the beginning of October, just after the second peak of movements (September). This feast strongly contributes to massive movements of small ruminants during the preceding months. Preliminary studies on Senegal livestock mobility data have shown a similar peak of movements around Tabaski. Our data are in good agreement with the volume expected by international agreements on livestock import around the period of Tabaski. However, to better assess the role of Tabaski, in particular when it falls during dry season, further analysis on mobility data during the first decade of 2000 should be conducted.

An important question is to know whether the bimodal spatio-temporal structure is a constant feature in the small ruminant trading network. Long-term follow-up demographic surveys were implemented in small ruminant farms from early 1980’s to early 2000’s in northern Senegal [58, 59]. Demographic analyses of these data revealed (i) a large and constant peak in male sheep off-take rate for the Tabaski, (ii) a trend for an increased off-take rate during the hunger gap, and (iii) a strong annual variability in off-take rates beside these trends, depending on rainfall and many other factors [45–47]. Therefore, further work is needed to investigate the temporal structure of the mobility network in the region.

Despite their limitation, these data provide an invaluable tool for targeting future field work, indicating markets and farming areas that are more active and central to the mobility network, and thus important to consider in disease surveillance and control programs.

## Supporting information

S1 Text Additional details. We provide further descriptions of the livestock mobility network and its variations along the year.
(PDF)
