# Supplementary material for: Towards the description of livestock mobility in Sahelian Africa: Some results from a survey in Mauritania
Source: PLoS One. 2018 Jan 24;13(1):e0191565. doi: 10.1371/journal.pone.0191565 (PMC5783398; doi:10.1371/journal.pone.0191565)
Supplement: S1 Text — We provide further descriptions of the livestock mobility network and its variations along the year. (PDF) [file pone.0191565.s001.pdf]

Towards the description of livestock mobility in Sahelian Africa: some results from a survey in Mauritania

Andrea Apolloni Galle Nicolas Caroline Coste
Ahmed Bezeid EL Mamy Barry Yahya Marius Gilbert
Renaud Lancelot Mohamed Baba Gueya Doumbia Baba
Ahmed Salem EL Arbi

January 12, 2018

Supplementary Information

In this section we summarize some of the characteristics of Mauritania and its mobility patters. Figure A shows the agro-ecological context of Mauritania. Mauritania is characterized by two large climatic area: hyper-arid in the North and arid in the rest of the country A(A). The aridity index, has been estimated as the ratio between the mean annual precipitation and the mean annual potential evaporation [3]and is a measure for the moisture availability for vegetation growth: the lower the value , the arider the area. Rainfalls data and precipitation have been collected for the last 30 years using satellite and ground measures (TAMSAT)[1].Rainfalls are concentrated in the rainy season (June-October). Figure A(B) show the variations of the yearly average precipitation volume from the average precipitation volume in the last 30 years. A negative value of the variation indicates a drier year, a positive value a rainier one. We notice that the last decade of the twentieth century has been particularly dry, in the recent years, 2011 and 2014 have been the driest. Finally Figure A(B) shows the distribution of cattle sheep and goats as estimated from the model developed by Nicolas et al. [2] using random forest techniques: each pixel in the figure is an estimate of the animal density by square kilometer. Supplementary Table A shows the distribution for the movements and volume according to species, transportation means, and either if is a national or an import/export movement. The table shows the net prevalence of small ruminants the leading role of exportation in animal trades. Moreover most of the animals are mostly moved walking on feet.

In Figure C, the monthly volume by species are analyzed in terms of the type of movement and the transportation mean. As stated before the majority of volume is exchanged internationally. This is particularly true for cattle and

		Movements	Volume (in millions)
	Total	2259	7.2
Species	Small Ruminants (Goats and Sheep)	1194	4.7
	Cattle	695	1.9
	Dromedaries	370	0.6
Transportation mean	Truck	598	1.3
	Feet	1661	5.9
National/International	National	1190	2.2
	Export	531	4.4
	Import	538	0.6

Table A: Characteristics of data collected. Volume indicates the number of heads, while movements indicates the number of movements between the locations

small ruminants trades, whilst for dromedaries the percentages of international and national volume are practically equal and constant during the year. The volume of national movements remains of the same order along the year for small ruminants and cattle. International movements for these two categories shows a peak during the first months of the year, during which the number of animals exported almost double or triply (Small Ruminants). In general, international movements are shorter than national one (Figure D(A)). This effect is mainly due to the way data have been collected: in fact respondents indicate the first locations abroad and not the final destination of the convoy. Moreover the drop in the average distance between April and June is mainly due to the fact that the majority of the transactions in this period are restricted in a narrow strip in the southern part of Mauritania. Finally we notice that truck transportation is used only for long distances compared to walking on foot (Figure D(C)).

We consider the 4 networks (Backbone, Frequent Intermediate and Occasional) whose links are defined based on their annual frequency. We studied the correlations and possible overlapping between couples of them. As construction links belonging to 2 different networks belong to 2 mutually exclusive sets and consequently the corresponding Jaccard index is null, thus the only possible comparison term for overlapping are nodes. As shown in Table B we see that the Jaccard index is low for all the possible couples, indicating a small overlapping between couple of networks. To estimate the correlation between networks, we use product-momentum procedure. To asses the significance of the correlation , we use a Quadratic Assignment Procedure test: we label-permute 1000 the networks and estimated the correlation among the permuted networks; we then evaluated the number of replicas whose correlations was larger than the true values experimental p-value. Table B shows results from the QAP test showing a non-significative correlation among all the couples of network

Supplementary Figure E shows the geographical distribution of the network in the two period of the year, independently of the species involved. In the first part of the year network is larger in terms of links and nodes. Most of

Networks Compared	Nodes' Jaccard Index	QAP tests results
Backbone-Frequent	0.20	-0.009 (p-value 0.689)
Backbone-Intermediate	0.33	-0.008 (p-value 0.731)
Backbone-Occasional	0.19	-0.009 (p-value 0.723)
Frequent-Intermediate	0.27	-0.007 (p-value 0.683)
Frequent-Occasional	0.34	-0.018 (p-value 0.708)
Intermediate-Occasional	0.22	-0.007 (p-value 0.699)

Table B: **Comparison of the 4 Networks** Since the 4 networks are not-overlapping , Jaccard Index for links is identical null and not shown. QAP test indicate the correlation estimate using QAP procedure and the corresponding p-value

the movements are concentrated in southern part across the border, but in the second part of the year, a larger fraction of the links are devoted to national trade. We notice that connections with Western Sahara are present through all the years.

Finally, Supplementary Figure F, shows a network representation of the 4 networks (Backbone, Frequent,Intermediate, Occasional), without any distinction in terms of species involved and period of the year. As we notice all the 4 networks present a large connected component, and few isolated nodes connected in couple or triple,except for the frequent one, whose giant component is made of a.

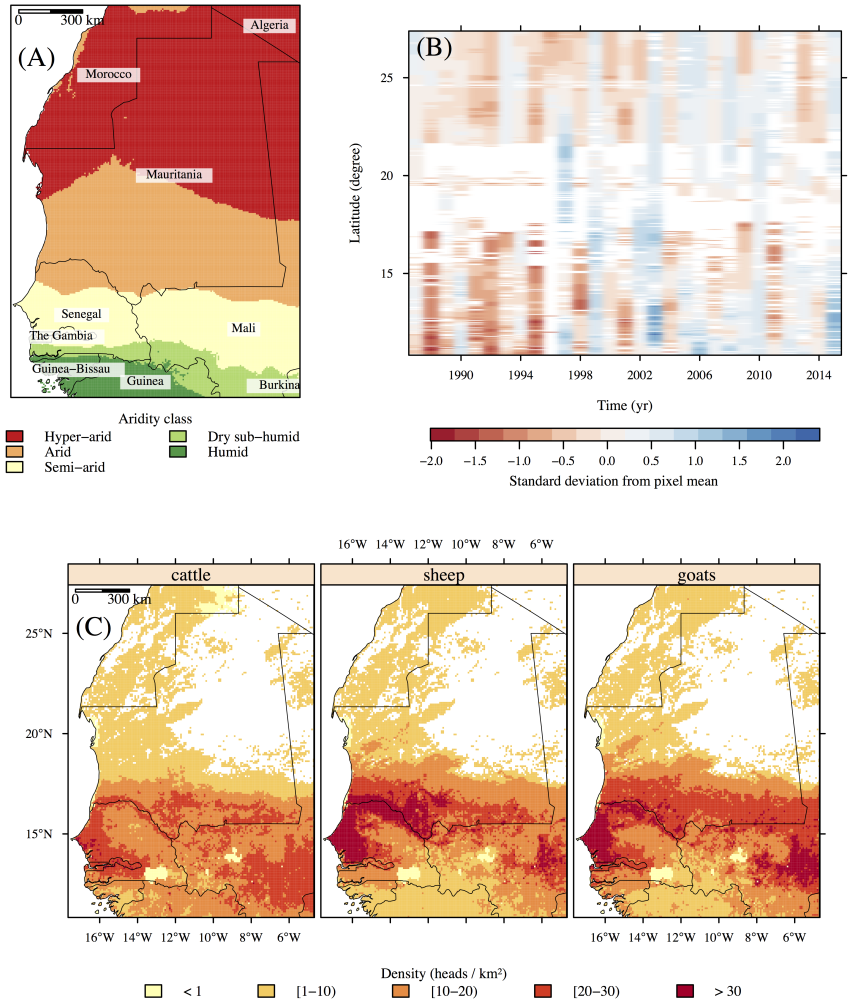

Figure A: Mauritania's Agro-ecological description (A) Aridity map; (B) Annual rainfall variability (standard deviation from the 1987-2015 mean at the pixel level); (C) Cattle and small ruminant density.

ORIGINE / DESTINATION

Nom de l'enquêteur : …………………………………………………………… **Lieu de l'enquête -** Région> localité : ……………………………………………………………………

Poste vétérinaire: ……………………..… **Coordonnées GPS** :……………………………….. **Date de l'enquête** : ……………..

**Mode d'élevage**	**Type**	**Espèce**	**Mode de déplacement**	**Fréquence**	**Période**
E – Extensif<br>I – Intensif<br>A – Autres	M - Marchés<br>C – Centre d'embouche<br>E - Elevage<br>*A* - Abattoirs-tueries<br>R – Zone de Repos	B – Bovins<br>C – Caprins<br>O – Ovins<br>Ca - Camelides	C – Camion<br>P – A pied	Q - Quotidien<br>H - Hebdomadaire<br>M - Mensuel<br>A - Annuel	Tabaski<br>Saison seche

Mode élevage	Origine	Type	Destination	Type	Espèce	Nombre approx.	Mode de transport	Date ou période	Fréquence	Durée trajet *jours*	Transit (étapes)	Débarquement O/N ?

Figure B: Copy of the form used for the mobility survey

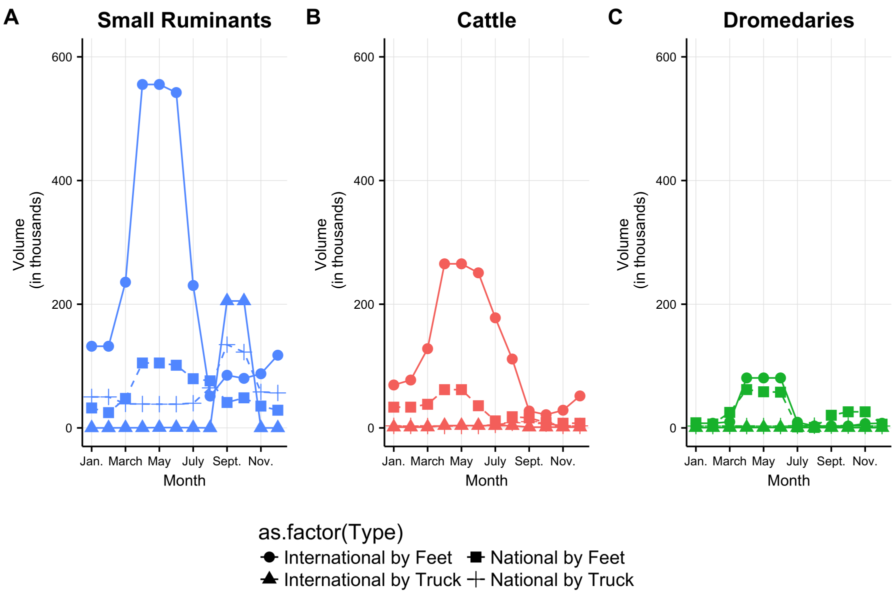

Figure C: Monthly volume of species moved according to type of movement. A) Small Ruminants B) Cattle C) Dromedaries. The line type and point shape correspond to the different types of movement by mean and destination (international or national)

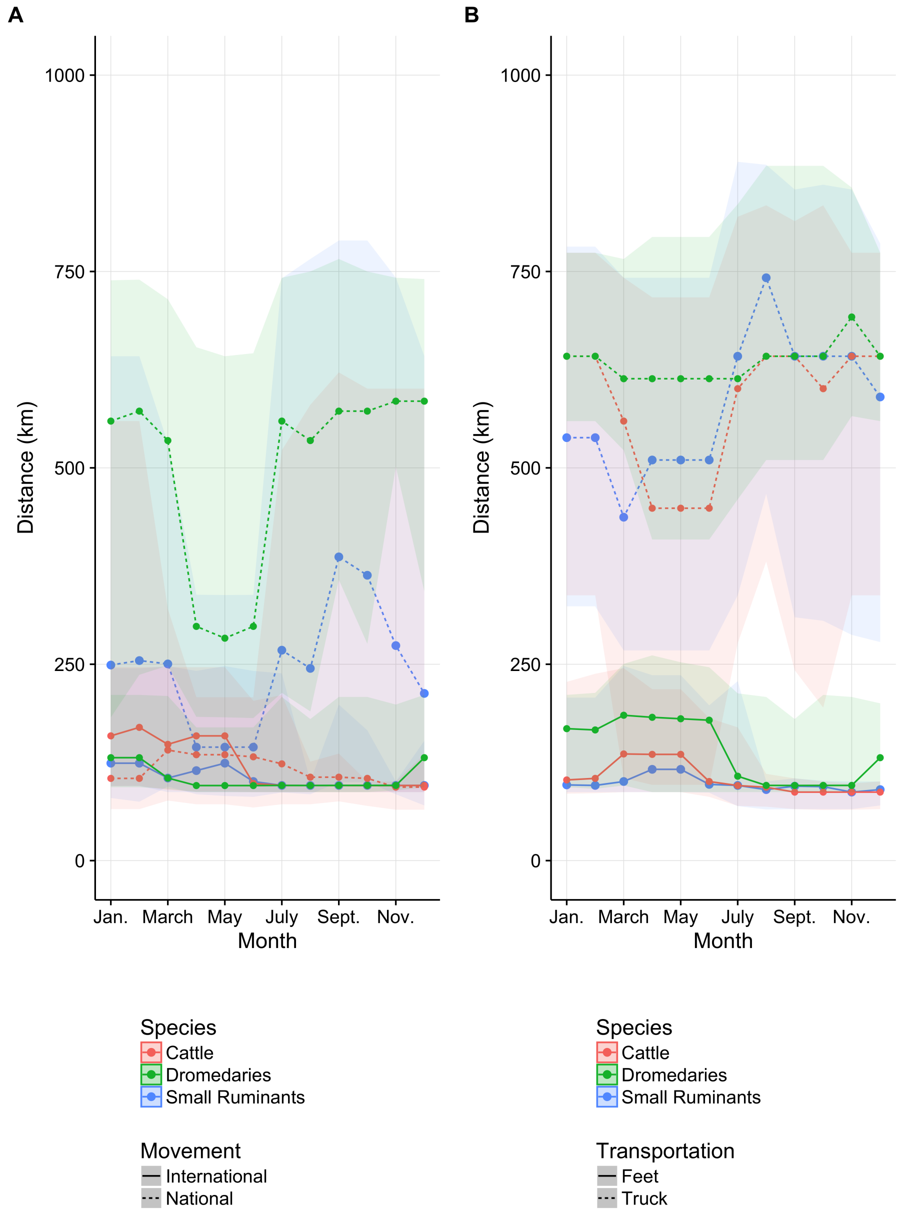

Figure D: Monthly distribution of herd's movement distance. Colors correspond to different species. A) Distribution for international and national movements B) Distribution by mean of transportation

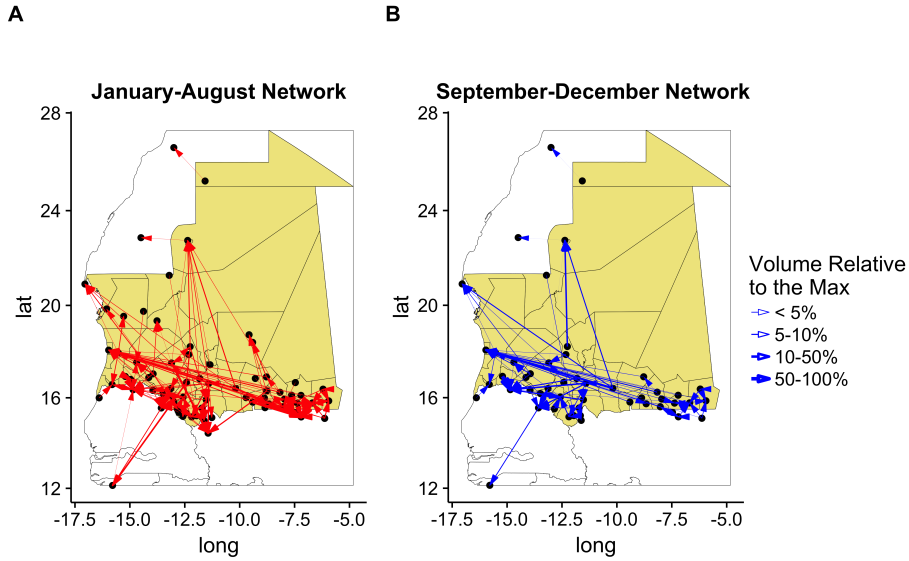

Figure E: Mobility network along the year; A Network during the first period B Network around Tabaski period.Thickness is proportional to volume moved along the connection, normalized to the maximum volume for the period of the year. Maps done using R version 3.3.1 (https://www.r-project.org).

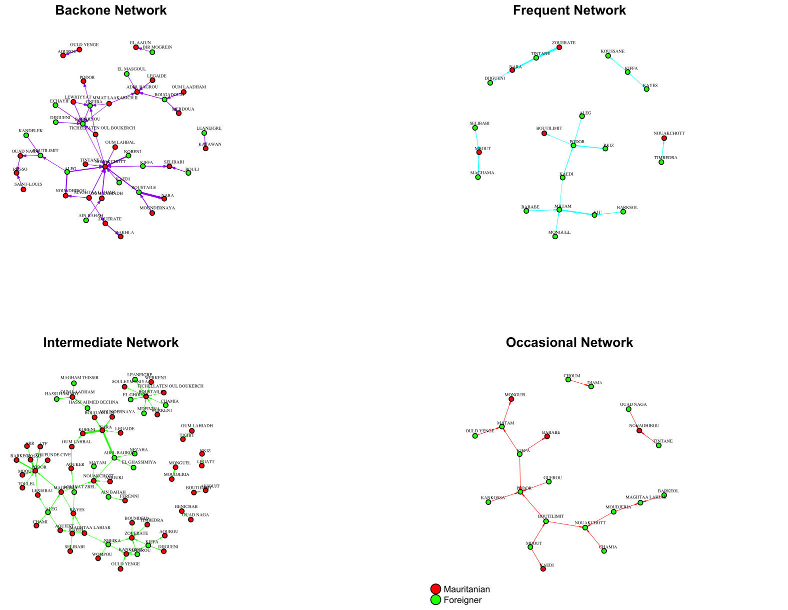

Figure F: Network representation of the 4 networks (Backbone, Frequent,Intermediate, Occasional). Nodes' colors are used to distinguish between Mauritanian and Foreigner locations, link width is proportional to the toal volume of animal traded on that direction

References

[1] Maidment, R. I., Grimes, D., Allan, R. P., Tarnavsky, E., Stringer, M., Hewison, T., Roebeling, R., and Black, E. The 30 year TAMSAT African rainfall climatology and time series (TARCAT) data set. *Journal of Geophysical Research: Atmospheres 119*, 18 (2014), 10,619–10,644.

[2] Nicolas, G., Robinson, T., Wint, G., Conchedda, G., Cinardi, G., and Gilbert, M. Using random forest to improve the downscaling of global livestock census data. *PLOS ONE 11*, 3 (03 2016), 1–16.

[3] Trabucco, A., and Zomer, R. *Global aridity index (global-aridity) and global potential evapo-transpiration (global-PET) geospatial database*. CGIAR Consortium for Spatial Information, 2009.
